# Ethnomedicinal knowledge of a marginal hill community of Central Himalaya: diversity, usage pattern, and conservation concerns

**DOI:** 10.1186/s13002-020-00381-5

**Published:** 2020-05-24

**Authors:** S. N. Ojha, Deepti Tiwari, Aryan Anand, R. C. Sundriyal

**Affiliations:** 1 G.B. Pant National Institute of Himalayan Environment, Kosi-Katarmal, Almora, Uttarakhand 263 643 India; 2 Department of Forestry and Natural Resources, HNB Garhwal University, (Garhwal), Srinagar, Uttarakhand 246174 India

**Keywords:** Ethnomedicinal plants, Traditional knowledge, Indigenous people, Ailments, Public health, Bageshwar, Uttarakhand

## Abstract

**Background:**

Indigenous communities use wild plants to cure human ailments since ancient times; such knowledge has significant potential for formulating new drugs and administering future health care. Considering this, the present study was undertaken to assess use value, diversity, and conservation concerns of medicinal plants used in traditional herbal care system of a marginal hill community in Bageshwar district of Uttarakhand in the Central Himalayan region of India.

**Methodology:**

Extensive surveys were made in 73 villages to gather information on the ethnomedicinal use of plant species used in the traditional herbal healing system. A total of 100 respondents were identified (30 herbal healers called *Vaidyas* and 70 non-healers/natives) and interviewed using semi-structured questionnaires, target interviews, and group discussion. Some important indices such as the use-value index (UV), relative frequency citation (RFC), cultural importance index (CI), and informant consensus factor (*F*_ic_) were calculated for the medicinal plants included in the present study.

**Result:**

It was recorded that the community uses a total of 70 species with 64 genera and 35 families for curing various ailments. Family *Lamiaceae* recorded the maximum number of medicinal plants. Twenty-one species used most extensively in the traditional health care system. The major parts of the identified plants used for the treatment of various ailments were root/rhizome and leaf. The most common methods used for the preparation of these plants were decoction and infusion. *Ocimum basilicum* L., *Cannabis sativa* L., *Citrus aurantifolia* (Christm) Sw., *Curcuma longa* L., and *Setaria italica* L. had the highest rate of use report. RFC value ranged between 0.03 and 0.91 with highest values for *Setaria italica*, *Zingiber officinale*, *Ocimum basilicum*, and *Raphanus sativus*. The traditional knowledge is passed verbally to generations and needs to be preserved for the future bio-prospecting of plants that could be a potential cure to any future disease.

**Conclusion:**

In recent years, the community has access to modern hospitals and medicinal facilities, although a considerable number still prefer medicinal plants for curing select ailments. It is suggested that these ethnomedicinal species need to be screened and evaluated further for their effectiveness for pharmacological activity. Also, significant efforts are required to conserve traditional knowledge and natural habitats of wild medicinal plants.

## Background

Medicinal plants have been utilized for the treatment of various diseases since ancient times, thus form an important element of aboriginal curative systems. The Indian *Rishis* first documented the use of medicinal plants in the form of Samhitas. *Charak Samhita* (1000–800 BC) and *Shushrut Samhita* (800–700 BC) by Maharshi Charak and and Maharshi Shashurut, respectively, are the baselines of the Indian Medicinal System. Maharshi Charak mentioned over 500 medicinal plants, out of which 340 plants used in the production of herbal medicine [1, 2]. AYUSH (i.e., Ayurveda, Unani, Siddha, and Homeopathy) is another traditional Indian health care system that is considered a great knowledge base in herbal medicines. Ayurveda reports over 2000 medicinal plant species, Siddha 1121 plant species, Unani 751 species, and homeopathy 422 species [3]. Nearly 70–80% population worldwide still relies on traditional medicinal systems for their primary health care because of their effectiveness, cultural preferences, and lack of modern health care alternatives [4, 5]. The global demand for herbal medicine continues to increase over the past few decades. The earlier studies stated that out of 250,000 flowering plants in the world, only less than 10% have been screened so far for their medicinal potency, and still, 90% remains unexplored [2]. In recent times, there is an increased interest regarding the use of the medicinal plants to develop new drugs and medicines for fulfilling the demand of a growing population [6–8]. Therefore, the information on plants of ethnomedicinal importance holds high potential. Uttarakhand Himalaya is a mountainous region in northern India that has a unique geography, rich biological resources, cultural heritage, and diverse climatic conditions which supports the highest number of medicinal plant species [9]. Over two-third population live in rural areas and depend on diverse natural resources to fulfill their need for food, fuel, fodder, timber, medicine, etc. Communities use a large variety of medicinal plants for treating diverse ailments [10, 11]. However, it is strongly being realized that the indigenous knowledge related to herbal medicines is continuously being eroded despite high significance to humanity. The subject needs further research such as documentation of potential medicinal species, analyzing their active constituents, clinical trials for validations, and developing new drugs and medicines [8–12]. Considering this, the present study was undertaken. We argue that sustainable management and conservation of medicinal plants can be achieved when information about their use for treating ailments and traditional herbal practices within particular areas are available. Such information is strongly desired to be preserved from being lost for the use of both the present and the future generations. For the purpose of this study, we selected marginal community and local herbal practitioners (Vaidyas) of Bageshwar district in Uttarakhand state in north India and documented ethnomedicinal plant diversity and traditional medicinal practices being used by them. Efforts were also made to scientifically validate and interpret the data using several indices such as relative frequency citation (RFC), use report (categorical and disease-based), cultural importance index (CI), and informant consensus index (Fic) so as to verify the homogeneity, importance, and the cultural similarity of the medicinal plants in communities. It is expected that the qualitative and quantitative information generated from the study will have immense utility for the conservation and sustainable utilization of medicinal plants as well as for managing the traditional health care system.

## Materials and methods

### Study area

**Fig. 1:**
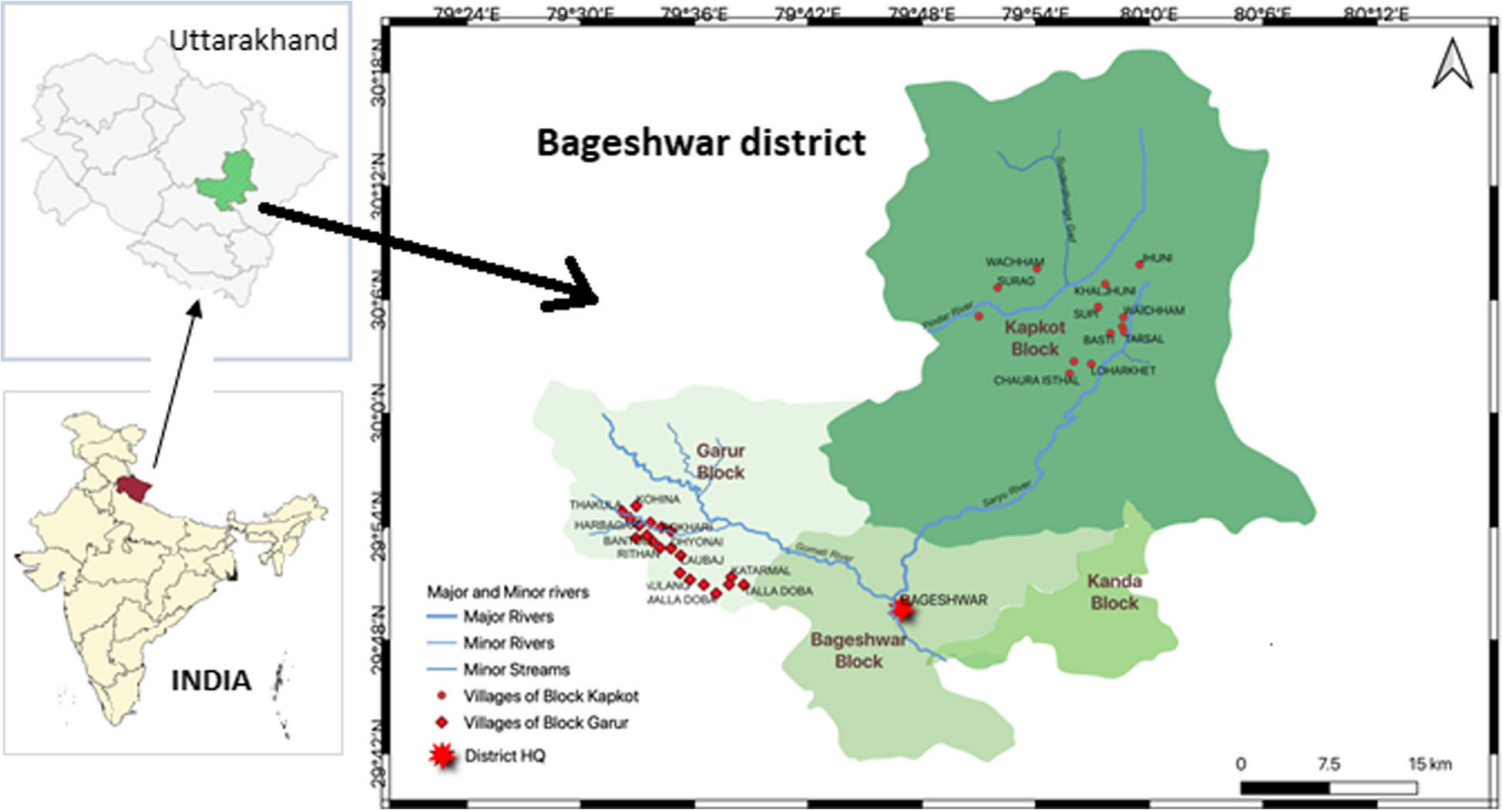
Study area and villages in Garur and Kapkot Bolcks of District Bageshwar, Uttarakhand, India

This study aimed to investigate the medicinal species used by the marginal hill community living in remote and high-altitude areas where medical health care facilities are not easily available. These practices are being used since eternity descended from the inherited knowledge of the locals and indigenous population of Uttarakhand. The study was carried out at Bageshwar district (geographical area 1687.8 km^2^) of Uttarakhand state and lies between latitudes 29° 42′ 40″ to 30° 18′ 56″ N and longitudes 79° 23′ to 80° 10′ E (Fig. 1). The district is situated on the confluence of Gomti river and Saryu river which is a tributary of Kali river. It is bounded by Almora district in the southwest, Chamoli district in the north and northwest, and Pithoragarh district in the east. Administratively, the district is divisible into four Tehsils, viz., Bageshwar, Kapkot, Kanda (Sub-tehsil), and Garur, and three blocks, viz., Bageshwar, Garur, and Kapkot. There are 947 revenue villages, out of which 874 villages are inhabited, and 73 villages are uninhabited. As per the 2011 census, the total population of Bageshwar district is 259,898 (male 48%, female 52%) with 96% living in the rural areas.

The community of the area is divided into 3 categories, viz., General, Scheduled Class (SC), Scheduled Tribe (ST), and majority of them involved in primary sector (agricultural activities), while some also work in secondary and tertiary sectors, such as private works, businesses, and government jobs. As such, the community is highly marginal with small and scattered land holdings, low production, and low income, therefore, highly dependent on natural resources. Male population out-migrates to earn better livelihoods that lead to continuous increase in fallow lands and culturable waste lands.

### Data collection

**Fig. 2:**
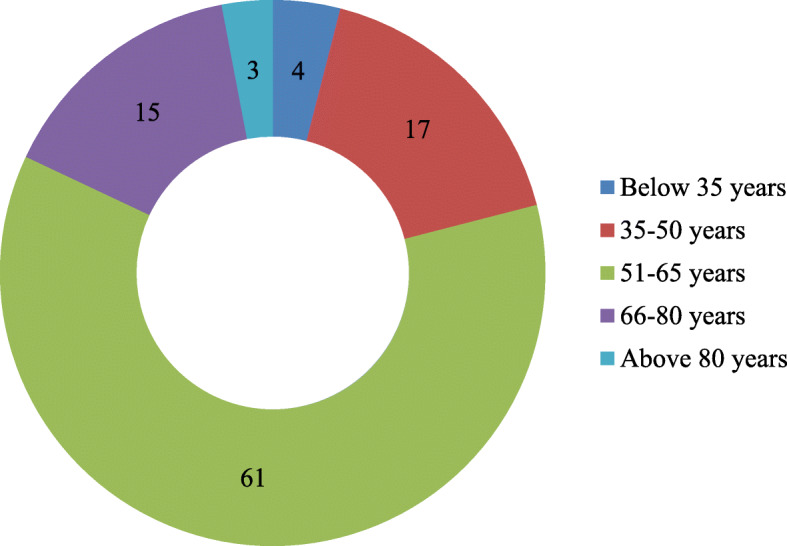
Age distribution of respondents

The study was conducted in 39 villages covering Garur-Ganga valley (23 villages) and Saryu valley (16 villages) of Garur and Kapkot Blocks during 2016–2018. To fulfill the objectives of the study, extensive field visits were made to gather information from traditional herbal healers (*Vaidyas*) and indigenous people using semi-structured questionnaires, target interviews, and visual interpretation through snowball methodology. A total of 100 respondents were randomly selected for the present study from both valleys, 37 being male and 63 female respondents. Of them, 30 were *Vaidyas* (male 19, female 11). Female informants were given preference in view of their dominance in villages. The age group of informants varied between 30 and 83 years, although most of them were between 50 and 65 years of age (Fig. 2). The questionnaire contains information about the ethnomedicinal plants with their local name, parts used, habit, ailment treated by medicinal plants, and mode of utilization of herbal formulation. Two general meetings and interviews were also organized at each valley with Vaidyas and natives. The documented medicinal plant species were validated for identification using available literature [13–16]. The specimens matched with the herbarium lodged in CCRAS-RARI, Tarikhet, Ranikhet, Uttarakhand (acronym RKT), which houses largest medicinal plant herbariums in northern India. A few generally available species were matched with the plant database of Centre for Socio-Economic Development deposited at G.B. Pant National Institute of Himalayan Environment (GBP-NIHE), Almora, Uttarakhand.

### The ethnobotanical analysis

The information on ethnomedicinal important species were recorded including the local names of the species, habit, their uses in different forms, the part used in the medical practice, mode of administration, and the condition of the plant (fresh or dry). The plants were classified into 12 main categories of ailments which were further divided into different respective subcategories based on disease and affected body part. The data were then statistically analyzed for different parameters. To enhance the indicative value of the ethnomedicinal study, suitable quantitative methods and approaches were used in the form of indices, such as relative frequency of citation (RFC), use report (based on illness, based on taxa), cultural importance (CI), and consensus factor of informants (Fic).

Use-report values (UR) provides information on the total number of reported uses for each species. It is similar to the use value of a species, but for use report, the number of events (interviews), the process of asking one informant on one day about the uses they know for one species, is one because the respondents were interviewed only once. And response use values are broken down by the number of uses reported for each plant species part.

Use-value index (UV) depicts the importance of each species for each informant and calculated by *UV* = ∑*U*/*N* formula where *U* is the number of uses quoted in each interview by *N*, number of informants. Use values are high when there are many useful reports for a plant representing its importance and come within reach to zero (0) when the use reports are low [17].

Relative frequency citation (RFC) index reveals the usage importance of a particular species used by different informants. The index is calculated by dividing the total number of informants referring to a particular taxon with the total number of informants (*RFC* = *FC*/*N*) where *FC* is the total number of informants that referred to the taxon, and *N* is the total number of informants [18].

Cultural importance index (CI) is estimated for each locality as the summation of Use-Report (UR) in every use category mentioned for a species in the locality divided by the total number of informants. This index provides an implication of the involvement of a particular taxon in the community, and a greater value signifies that a particular is widely distributed among communities. A null value indicates non-existence of the species in the area. CI is calculated as *CI = UR/N* where *UR* is the total number of use reports for each species in every category of illness mentioned, and *N* is the total number of informants [19].

Informant consensus factor (*Fic*) is used to test the consistency of information knowledge in treating a particular illness category. The values obtained are near one (1) for well-defined selection criteria in the community and/or if the information is exchanged between the informants. A value approaching zero (0) represents that the plants are chosen randomly, and/or there is no information exchanged between the communities about their use. *Fic* is calculated as *Fic* = (*Nur − Nt*)/(*Nur −* 1), where *Nur* refers to the number of use reports for a particular use category, and *Nt* refers to the number of taxa used for a particular use category by all informants [20].

## Result and discussion

### Ethnomedicinal uses of plants and mode of practice

**Fig. 3:**
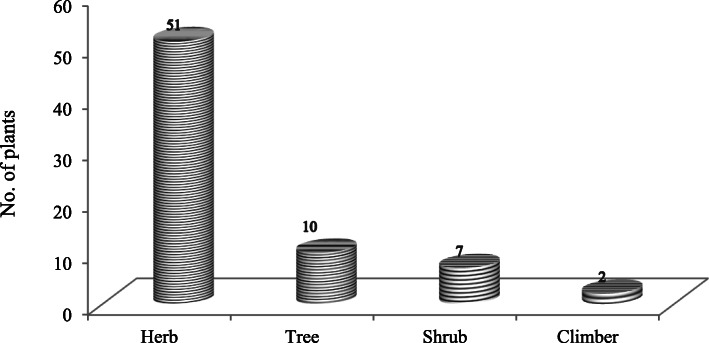
Distribution of medicinal plants in different life form

The residents of different age groups were surveyed to assess the ethnomedicinal uses of plant species (Fig. 2). The survey revealed that a total of 70 medicinal plant species varying from 35 families and 64 genera have been used by the inhabitants of 39 villages for different (Table 1). Family *Lamiaceae* recorded maximum species (8) followed by *Asteraceae* (6 species), *Fabaceae* (5 species), *Rosaceae* (4 species), and *Apiaceae*, *Liliaceae*, *Ranunculaceae*, *Rutaceae*, and *Zingiberaceae* (3 species each). The remaining families were represented with just one or two species. Almost all the species are widely used by the community. Of the total documented medicinal plant species, the herbaceous habit (51 species) was the most dominant life form, followed by the tree (10), shrub (7), and climbers (2 species) (Fig. 3).

**Table 1:** Quantitative enumeration of ethnomedicinal plants used by marginal hill community of District Bageshwar

Botanical name	Local name	Voucher/ident. no.	Habit	Part used	Popular ailment uses (group and categories)	Used in	Preparation	FC^**a**^	RFC^**b**^	UR^**c**^	UR^**d**^	CI^**e**^
Family: *Alliaceae*												
*Allium sativum* L.	Lasan	GBPCSED1	H	B	Skeleton and muscles—joint pain (arthritis)	Hu	O	59	0.59	59	59	0.59
**Family:***Apiaceae*												
*Angelica glauca* Edgew.	Gandaraini	RKT 27789	H	Rt	Gastrointestinal—stomach ache, vomiting	Hu	Po	44	0.44	35	89	0.89
					Other—spices and condiment, herbal tea		Co, Inf			54		
*Centella asiatica* L.	Brahmi	RKT 28186	H	L	General health care - Headache	Hu	Po	28	0.28	28	28	0.28
*Coriandrum sativum* L.	Dhaniya	RKT 28118	H	Sd	Antidote—against poison	C	Em	36	0.36	36	36	0.36
**Family:***Araceae*												
*Acorus calamus* L.	Bojh/Buch	RKT 27965	H	Rh	Skeleton and muscles—sprain, inflammation	Hu	Pw, O	55	0.55	21	74	0.74
					Other—insect repellent	I	Da			53		
**Family:***Asteraceae*												
*Ageratina adenophora* (Spreng.) King & H. Rob.	Nargadiya/Pagaljhad	RKT 22106	H	L	Dermatological—cuts and wounds	Hu	Po	80	0.8	80	80	0.8
*Artemisia martima* L.	Pati/Titpati	RKT 23793	H	L	Dermatological—cuts and wounds, skin ailments	Hu	Po	55	0.55	77	77	0.77
*Saussurea costus* (Falc.) Lipsch.	Kut/Kuth	RKT 28203	H	Rt	General health care—fever	Hu	Pw	28	0.28	27	64	0.64
					Respiratory—cough		Pw			8		
					Gastrointestinal—stomach ache, dysentery		De			29		
*Taraxacum officinale* Weber.	Dudhil	RKT 27817	H	L,Rt	Antidote—snake bite	Hu	In, Po	50	0.5	13	39	0.39
					Other—to increase lactation in mulching animals	C	Inf			26		
*Tegetus erecta* L.	Hazari	GBPCSED2	H	L	General health care—fever, ear infection	Hu	Po	51	0.51	46	61	0.61
					Dermatological—wounds		Po			15		
**Family:***Berberidaceae*												
*Berberis asiatica* Roxb. ex DC	Kilmori	RKT 22109	S	Rt	General health care—fever	Hu	Pw	42	0.42	13	54	0.54
					Circulatory—diabetes		Pw			41		
**Family:***Boraginaceae*												
*Cynoglossum zeylanicum* Thunb. Ex Lehm.	Chtkura	RKT 22969	H	Rt	Dermatological—boils	Hu	Da	54	0.54	54	54	0.54
**Family:***Brassicaceae*												
*Rephanus sativus* L.	Mooli	RKT 27049	H	WP	Hepatic health cure—jaundice	Hu	Co	87	0.87	87	87	0.87
**Family:***Cannabaceae*												
*Cannabis sativa* L.	Bhaang	GBPCSED3	H	Sd, L	Gastrointestinal—purgative and laxative, carminative, constipation, stomach ache	Hu	In	63	0.63	46	94	0.94
					Antidote—insect bite		Da			5		
					Other—warm effect in winters		In, Co			43		
**Family:***Caryophyllaceae*												
*Drymaria cordata* (L.) Willd. ex Schult	--	RKT 19989	H	WP	Respiratory—cough	Hu	In	19	0.19	7	7	0.07
*Silene vulgaris* (Moench) Garcke	Pyankura	GBPCSED4	H	WP	General health care—fever	Hu	De	15	0.15	4	17	0.17
					Gastrointestinal—removal of *Ascaris* (antiparisitic) locally known as *juga*		De			13		
**Family:***Combretaceae*												
*Terminalia chebula* (Gaertner) Retz.	Harar	RKT 15469	T	Fr	Gastrointestinal—purgative and laxative, carminative, constipation, digestive problems, diarrhea	Hu	Pw, Po	12	0.12	64	64	0.64
**Family:***Cucurbitaceae*												
*Momordica charantia* L.	Karela	RKT 27529	Cl	Fr	Circulatory—diabetes	Hu	Co, In	39	0.39	39	39	0.39
**Family:***Dioscoreaceae*												
*Dioscorea deltoidea* Wall.	Genthi	RKT 27301	Cl	Fr (Atu)	Respiratory—cough and cold	Hu	Co	32	0.32	32	32	0.32
**Family:***Ericaceae*												
*Rhododendron arboreum* Smth	Burans	RKT 27288	T	F	Hepatic health cure—liver complaints, tonic	Hu	De	47	0.47	64	64	0.64
**Family:***Euphorbiaceae*												
*Emblica officinalis* Gaertn.	Aanwla	RKT 21022	T	Fr	Circulatory—diabetes	Hu	In	35	0.35	8	85	0.85
					Gastrointestinal—purgative and laxative, carminative, stomach ache		In			54		
					Respiratory—cough		In			6		
					Other—source of vitamin “C”		In			17		
*Euphorbia prolifera* Ehrenb. Ex. Boiss	Dudhiya, Maikuri	RKT 29216	H	WP	Other—insect repellent	I	Da	7	0.07	7	7	0.07
**Family:***Gentianceae*												
*Swertia angustifolia*	Chiraita	RKT 25110	H	WP	General health care—fever	Hu	In	37	0.37	19	24	0.24
Buch.-Ham. ex D.Don					Dermatological—skin ailments		In			5		
**Family:***Fabaceae*												
*Glycine max* (L.) Merri	Kala Bhatt	RKT 15664	H	Sd	Hepatic health cure—jaundice	Hu	Co	84	0.84	84	84	0.84
*Microtyloma uniflorum* (Lam) Verdc.	Gahat/Kulthi	GBPCSED5	H	Sd	Urinogenital disorder—stone	Hu	Co	69	0.69	69	69	0.69
*Trifolium repens* L.	Chalmoda	RKT 26479	H	L	General health care—headache	Hu	Po	44	0.44	18	22	0.22
					Dermatological—skin disease of dogs-Luta	C	Po			4		
*Trigonella foemun-graecum* L.	Maithi	RKT 28507	H	L, Sd	Circulatory—diabetes	Hu	Inf	31	0.31	11	61	0.61
					Gastrointestinal—carminative, obesity, indigestion, constipation		Inf			47		
					Skeleton and muscles—joint pain		Inf			3		
*Vigna mungo* L. (Fabaceae)	Mass, Urad	RKT 27199	H	Sd	Skeleton and muscles—fracture	Hu	In	61	0.61	61	61	0.61
**Family:***Lamiaceae*												
*Ajuga bracteosa* Wall. ex Benth.	Ratpatia	RKT 25182	H	WP	General health care—fever	Hu	De	55	0.55	53	72	0.72
					Gastrointestinal—constipation					16		
					Urinogenital—diuretic					3		
*Ajuga parviflora* Benth.	Ratpatia	RKT 26408	H	Rt	General health care—fever, throat infection in animal (*Galghotu*)	Hu and C	De, Em	56	0.56	58	87	0.87
					Gastrointestinal—constipation, stomach ache	Hu	De, In			25		
					Urinogenital—stone		De			4		
*Leucas lanata* Benth	Nirasi Jhad	RKT 29214	H	L	Respiratory—cough	Hu	De	80	0.8	80	80	0.8
*Mentha arvensis* L.	Pudina	RKT 4355	H	L	Gastrointestinal—stomach ache, vomiting	Hu	De	43	0.43	50	50	0.5
*Micromeria biflora* Benth.	--	RKT 22949	H	WP	General health care—fever	Hu	De	6	0.06	6	6	0.06
*Ocimum basilicum* L.	Tulsi	RKT 19325	S	L, Sd	General health care—fever	Hu	De	88	0.88	33	97	0.97
					Respiratory—cough and cold		De			41		
					Other—herbal tea, warm effect in winters		De			23		
*Origanum vulgare* L.	Van Tulsi	RKT 29244		L, Rt	General health care—fever	Hu	De	31	0.31	15	71	0.71
					Respiratory—cough and cold		De			18		
					Dermatological—wounds		Em			29		
					Other—herbal tea		Inf			9		
*Thymus serpyllum* L.	Van-ajwayan	RKT 27966	H	WP	Skeleton and muscles—joint pain	Hu	Em	18	0.18	3	14	0.14
					Respiratory—asthma		Em			3		
					Gastrointestinal—digestive and stomach problems		Em			4		
					Other—spices and condiments		Da			4		
**Family:***Liliaceae*												
*Asparagus racemosus* Willd.	Keruwa	RKT 28055	S	Rt	Immuno-regulatory—stimulant	Hu	Pw	46	0.46	15	65	0.65
					Hepatic health cure—tonic		Pw			39		
					Gastrointestinal—stomach ache		De			11		
*Polygonatum cirrhifolium* (Wall.) Royle	Maha-Meda	RKT 26144	H	WP	Hepatic health cure—tonic	Hu	De	21	0.21	13	34	0.34
					Dermatological—cuts and wounds		Po			14		
					Circulatory—blood purifier		Co			7		
*Polygonatum verticillatum* L.	Meda	RKT 25894	H	Rt	Gastrointestinal—carminative	Hu	In	15	0.15	8	19	0.19
					Dermatological—wounds		Po			11		
**Family:***Moraceae*												
*Ficus palmata* Forsk.	Bedu	RKT 28094	T	Lt	Dermatological—cuts and wounds	Hu	Da	48	0.48	39	39	0.39
*Ficus roxburghii* Wall.	Timul	GBPCSED6	T	Fr	Gastrointestinal—acidity, carminative	Hu	Co	26	0.26	45	48	0.48
					Circulatory—blood pressure		Co		0	3		0
**Family:***Myricaceae*												
*Psidium guajava* L.	Amrood	GBPCSED7	T	L	General health care—mouth blisters (astringent)	Hu	In	12	0.12	12	12	0.12
**Family:***Orchidaceae*												
*Dactylorhiza hatagirea* (D.Don) Soo	Salmpanja/Hattajari	RKT 26089	H	Rt	Circulatory—bleeding	Hu	De	17	0.17	17	34	0.34
					Dermatological—wounds		Po			17		
**Family:***Plantaginaceae*												
*Plantago ovate* Forsk.	Isabgoal	RKT 1899	H	Sd	Gastrointestinal–constipation, digestive problems, diarrhea	Hu	In	74	0.74	83	83	0.83
*Plantago lanceolata* L.	Jonkpuri	RKT 8154	H	Rt	Gastrointestinal—removal of stomach worm of domestic animals	C	In	43	0.43	43	43	0.43
**Family:***Poaceae*												
*Hordium vulgare* L.	Jau	RKT 26630	H	Sd	Hepatic health cure—warm and nutritive effect	Hu	Co	46	0.46	46	63	0.63
					Dermatological—burns		O			17		
*Setaria italica* L.	Kouni	RKT 7389	H	Sd	Dermatological—measles and chicken pox	Hu	Co	91	0.91	91	91	0.91
**Family:***Podophyllaceae*												
*Podophyllum hexandrum* Royle	Van-Kakri	RKT 27764	H	Fr, Rt	Dermatological—wounds	Hu	Po	19	0.19	19	19	0.19
**Family:***Polygonaceae*												
*Rheum emodi* Wall.	Dolu	RKT 27793	H	Rt	General health care—fever	Hu	De	31	0.31	15	42	0.42
					Dermatological—wounds		Po			27		
**Family:***Punicaceae*												
*Punica granatum* L.	Darim	RKT 28845	T	Fr	Respiratory—cough and cold	Hu	In	59	0.59	49	71	0.71
					Hepatic health cure—anemia		De			10		
					Other—source of vitamin “C”		De, In			12		
**Family:***Ranunculaceae*												
*Aconitum heterophyllum* Wall.	Atis	RKT 29008	H	Rt	General health care—fever	Hu	Pw	34	0.34	34	51	0.51
					Gastrointestinal—vomiting		In			17		
*Ranunculus repens* L.	Aingadua	GBPCSED8	H	Rt	Dermatological—boils	Hu	Po	21	0.21	21	27	0.27
					Gastrointestinal—intestinal pains (*NasPalatana*)		In			6		
*Thalictrum foliosum* DC.	Uppankat hi/Mamira	RKT 29204	H	WP	Ophthalmic—eye infection (white dot-cataract)	Hu	Inf	4	0.04	9	21	0.21
					Other—insect repellent	I	Da			12		
**Family:***Rosaceae*												
*Duchesnea indica* (Andrews) Focke	Van Kafal	GBPCSED9	H	L	Dermatological—burns and removal of burn scars	Hu	Po	3	0.03	3	3	0.03
*Prunus persica* Stokes.	Aaru	RKT 26465	T	L	General health care—Headache	Hu	Po	6	0.06	6	6	0.06
*Rosa moschata* Hermm.	Kunja	RKT 28695	S	L, F	Dermatological—cuts and wounds, boils	Hu	Po	9	0.09	27	32	0.32
					Ophthalmic—eye diseases		Ste			5		
*Rubus ellipticus* Smith.	Hisalu	RKT 29240	S	Rt	General health care—fever	Hu	De	9	0.09	9	18	0.18
					Gastrointestinal—stomach ache		De			9		
**Family:***Rubiaceae*												
*Rubia cordifolia* L.	Manjistha	RKT 27933	H	Rt	General health care—fever	Hu	De	27	0.27	23	23	0.23
**Family:***Rutaceae*												
*Citrus aurantifolia* (Christm) Sw.	Kagji	GBPCSED10	T	Fr	General health care—headache	Hu	De	38	0.38	20	94	0.94
	Nimboo				Gastrointestinal—constipation, weight loss		De			23		
					Respiratory—cold		De			19		
					Other—herbal tea, source of vitamin “C”		De			32		
*Citrus hystrix* DC.	Jamer/Jamir	GBPCSED11	T	Fr	Gastrointestinal—removal of *Ascaris* (antiparisitic) locally known as *juga*	Hu	In	38	0.38	27	50	0.5
					Respiratory—cold		In			7		
					Antidote—against poison	C	Em			16		
*Zanthoxylum armatum* DC	Timoor/Timuru	RKT 28615	S	Sd	General health care—toothache	Hu	In	61	0.61	21	77	0.77
					Respiratory—cough and cold		In			19		
					Gastrointestinal—carminative		In			6		
					Other—spices and condiments		In			31		
**Family:***Saxifragaceae*												
*Bergenia ciliata* (Haw) Sternb	Silphora	RKT 25124	H	Rt	Urinogenital—urinary infection, stone	Hu	Inf, Pw	51	0.51	61	61	0.61
**Family:***Scorphulariaceae*												
*Picrorhiza kurrooa* Royle.	Kutki	RKT 27765	H	Rt	General health care—fever	Hu	In	53	0.53	53	80	0.8
					Gastrointestinal—abdominal pain		In			27		
*Verbascum thapsus* L.	Akalveer	RKT 27890	H	WP	Dermatological—boils	Hu	Po	63	0.63	17	42	0.42
					Other—to increase lactation in milching animals		Da			25		
**Family:***Urticaceae*												
*Urtica dioica* L.	Shishun/Bichhu ghas	RKT 22903	S	L	Skeleton and muscles—joint pain	Hu	Da	37	0.37	31	52	0.52
					Hepatic health cure—warm and nutritive effect	Hu	Co			21		
**Family:***Violaceae*												
*Viola betonicifolia* J.E. Smith	Garurjadi/garurabuti	GBPCSED12	H	WP	Antidote—snake bite	Hu	Po	12	0.12	13	13	0.13
*Viola canescens* Wall. Ex Roxb	Gulovansh	RKT 17561	H	WP	Other—to increase lactation in milching animals	C	Da	29	0.29	29	29	0.29
**Family:***Zingiberaceae*												
*Curcuma longa* L.	Haldi	RKT 5970	H	Rh	General health care—internal injury	Hu	De	78	0.78	39	91	0.91
					Dermatological—cuts and wounds, cosmetics		Da			36		
					Respiratory—cough		De			16		
*Hedychium spicatum* Buch. Ham. ex Smith.	Van Haldi	RKT 24059	H	Rh	Gastrointestinal—intestinal problems, purgative and laxative, carminative	Hu	Pw	13	0.13	30	52	0.52
					Respiratory—cough		Pw			8		
					Dermatological—cosmetics, anti-lice	Hu & C	Pw			14		
*Zingiber officinale* Rosc.	Adrak	RKT 5921	H	Rh	Respiratory—Cough and cold	Hu	Em	89	0.89	89	89	0.89

*Atu* aerial tuber, *B* bulb, *C* cattle, *Cl* climber, *Co* cooking, *De* decoction, *Da* direct application, *Em* emulsion, *F* flower, *Fr* fruit, *H* herb, *I* insect, *Inf* infusion, *In* ingestion, *hr* hour, *Hu* human, *L* leaves, *Lt* latex, *O* ointment, *Po* poultice, *Pw* powder, *Rh* rhizome, *Rt* root, *S* shrub, *Sd* seed, *Ste* steam, *T* tree, *WP* whole plant

^a^Use citation of taxa (the no. of informants that referred the taxon)

^b^RFC = FC/*N*, where *N* is the total no. of informants

^c^Use reports of the taxon by ailment category

^d^Use reports of the taxon

^e^CI = UR/*N*_t_, where *N*_t_ is the total no. of reported taxa

**Fig. 4:**
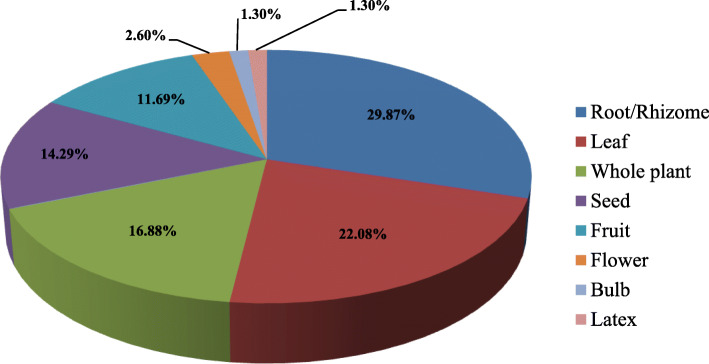
Plant part used in preparation of medicine

**Fig. 5:**
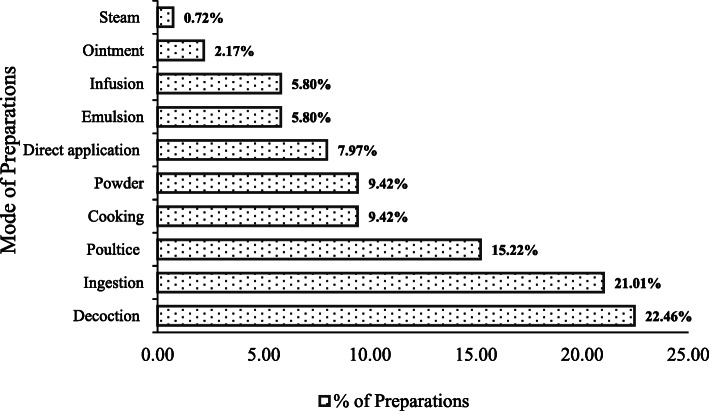
Processing of plant parts in preparation of medicine

It was interesting to note that nearly 70% population still use prescription of *Vaidyas* for common ailments, although the *Vaidyas* were having an age of > 50 years. The diseases cured by *Vaidyas* comprised fever, stomach problems, cough, cold, headache, etc. The most common plant parts used were root/rhizome, followed by leaf, whole plant, seeds, fruits, flower, and bulb and latex (Fig. 4). The collection of plant parts was very selective keeping into consideration the time of collection, plant matureness, and quantity of use thus ensuring a conservation approach. *Vaidyas* comprised sound knowledge and a species-specific method of preparing drugs to cure various ailments (Table 2)**.** Making decoction and ingestion was the most common mode of plant part use (Fig. 5). Poultice and cooking were also favored for many medicinal plants. Another mode of application includes cooking and making into powder (9.42%), direct application (7.97%), emulsion and infusion (5.80%), and ointment (2.17%) (Fig. 5). A decoction is the most commonly used method to cure ailments in traditional herbal systems [21–25]. It is considered to extract all potential bioactive compounds after heating [26]. The pleasant taste of the herbal drug can be attuned by adding together honey or sugar [27]. Ingestion and poultice were also common after crushing and/or mixing the plant parts with some solvent for application as paste and Band-Aid. In skeletal, muscle, and dermatological issues, application of plant parts as ointment was most prevalent.

**Table 2:** Bio-processing of medicinal plants of District Bageshwar

Scientific name	Mode of administration
*Aconitum heterophyllum* Wall*.*	Dry root powder (1 TS) taken orally with boiled water twice a day for 2–3 days against fever; 1–2 roots chewed to control vomiting.
*Acorus calamus* L.	Root powder mixed with grains used as insect repellent; 3–4 dry roots heated with mustard oil applied on the sprain and inflammatory region.
*Ageratina adenophora* (Spreng.) King & H. Rob	Leaf paste prepared from 100 g fresh leaf twigs applied on affected parts for early healing.
*Ajuga bracteosa* Wall. ex Benth.	Juice of whole plant (10–20 ml) taken twice a day for 2–3 days.
*Ajuga parviflora* Benth.	Decoction prepared from 100 g fresh or dried roots with water given 3–5 TS orally in fever, stomach ache, and constipation for 5 days; this decoction taken orally in empty stomach regularly for stone; 1–2 leaves chewed on empty stomach for gastric problem; decoction of whole plant (5–8) crushed with red chili (3) and 100 g Jiggery (*Gur*) given twice a day for 2–3 days to treat throat infection in domestic animals.
*Allium sativum* L.	Paste prepared from 5–7 spilled bulb heated with 20 ml mustard oil, massage on joints.
*Angelica glauca* Edgew.	Root powder (50 g) mixed with 100 ml water used to control vomiting and stomach ache; rhizomes are used as spices and condiments and tea (as flavor).
*Artemisia martima* L*.*	Juice (5–10 ml) of fresh leaf applied on the affected area.
*Asparagus racemosus* Willd.	Root decoction (100 g) prepared in water given to cure stomach ache (5 ml for adult, 1 TS for children) for 3–5 days, one palm full root powder taken with water as stimulant and tonic.
*Berberis asiatica* Roxb. ex DC	Root powder (100–150 g) taken with warm water given twice a day for 3 days against fever; fresh or dried roots soaked in water overnight, filtered, and taken orally to cure diabetes in empty stomach.
*Bergenia ciliata* (Haw) Sternb.	Fresh or dried roots (50–100 g) socked overnight and filtered, taken orally in morning for kidney stone. Root powder (50 g) taken with water twice a day for urinary infection.
*Cannabis sativa* L.	Grinded seeds cooked with some local vegetables (e.g., *Colacasia esculanta*, *Brassica oleracea*) for warm effect; broiled seeds are grinded with salt and green chili to prepare salt (*Pahadi namak*). Broiled seeds grinded with *Punica garnatum* mixed with green leaves of coriandum, green chili, salt, and sugar to prepare *Chatni*; fresh leaves crushed with 3–5 seeds of black pepper and applied on insect bite.
*Centella asiatica* L.	Fresh leaf paste is applied on forehead.
*Citrus aurantifolia* (Christm) Sw.	Juice extracted from fruit mixed with 1 TS honey, and 50 ml water taken orally in empty stomach for constipation and weigh loss; lemon tea used in fever and cold.
*Citrus hystrix* DC.	Fruit juice given orally (1 TS) to children for removal of *Ascaris*; cough and cold 10 ml thrice a day; fruit juice with mentha leaves (100 g) and coriander seeds made into paste given to domestic animals against poison.
*Coriandrum sativum* L.	Seed (80–100 g) paste mixed with 1–2 l processed curd (*Mattha*) is given to domestic animals against poison for 2–3 days.
*Curcuma longa* L.	Haldi powder (5 g) mixed with a full glass of warm milk for internal injury; paste of rhizome applied on cuts and wounds.
*Cynoglossum zeylanicum* Thunb. Ex Lehm.	Fresh or dried root paste applied on the affected parts.
*Dactylorhiza hatagirea* (D.Don) Soo.	Decoction of 100 g root with water taken orally (10–15 ml) twice a day for excessive bleeding; root paste applied on wounds.
*Dioscorea bulbifera* L.	Broiled fruit and cooked vegetable.
*Drymaria cordata* (*L*.)	Juice of aerial parts (2–4 drops) taken orally for 2–3 days.
*Duchesnea indica* (Andrews) Focke	Leaf paste is regularly applied on affected part.
*Emblica officinalis* Gaertn.	Fresh fruits are chewed regularly to control diabetes; dried fruits (3–5) boiled with water, filtered, and taken orally against cough and stomach ache; fresh and processed fruits are source of vitamin “C.”
*Euphorbia* sp.	Whole plant (50–100) mixed with FYM.
*Ficus palmata* Forsk.	Milky latex applied on cuts and wounds.
*Ficus roxburghii* Wall.	Fresh fruits are cooked as vegetable.
*Glycine max* (L.) Merri	*Bhatt ka Jaula* (an indigenous dish) is prepared from paste of seeds (soaked overnight) and cooked with rice in an iron vessel *Kadahi*.
*Hedychium spicatum* Buch. Ham. ex Smith.	Dried rhizome powder (2–3 g) taken with hot water once a day; paste of fresh rhizome used as anti-lice.
*Hordium vulgare L.*	*Sattu* prepared from 200 g broiled seeds mixed with 100 g jaggery (*Gur*) and 100 g *Ghee* for warm and nutritive effect; 50 g broiled seeds heated with 40 ml mustard oil applied on burns.
*Leucas lanata* Benth	Leaf juice with 3–5 drops of breast milk taken orally twice a day for 1 week.
*Mentha arvensis* L.	Leaves (100 g) boiled with water and filter, the filtrate (50 ml) given orally twice a day.
*Micromeria biflora* Benth.	Juice of whole plant with water (1–2 times in a day).
*Macrotyloma uniflorum* (Lam) Verdc.	*Gahat ka Ras* (an indigenous dish) prepared by 150 g seeds cooked with water (1 l) until the volume reduced (100 ml) and taken regularly.
*Momordica charanti* L*.*	Vegetable and juice (50 ml) of fresh fruit taken regularly.
*Ocimum basilicum* L.	Decoction of 100 g leaves and seeds, zinger (50 g), 5 seeds black paper with 150 ml water taken orally 2–3 times a day for fever, cough, and cold; aerial part used to make herbal tea.
*Origanum vulgare* L.	Decoction of 100 g fresh and dried leaves with water taken orally (10 ml) for a week in cough, cold, and fever; root paste applied on wounds.
*Picrorhiza kurrooa* Royle*.*	Decoction of 50 g root with water taken orally against fever and abdominal pain for 5–7 days.
*Plantago ovate* Forsk.	Seeds (10 g) soaked overnight or consumed directly with water twice a day for 30 days against constipation and digestive problems; Isabgoal (15 g) mixed with 10 TS fresh curd taken after meal for diarrhea.
*Plantego lanceolata* L*.*	Paste of roots (100 g) given to domestic animals.
*Podophyllum hexandrum* Royle	Root paste applied on wound.
*Polygonatum cirrhifolium* (Wall.) Royle	Small pieces of tuber (8–10) soaked in water for overnight, taken in empty stomach for weakness, and develop immunity; cooked green leaves eaten as blood purifier; root paste applied on cuts and wounds.
*Polygonatum verticillatum* L. All	Root powder (50 g) is taken with warm water in gastric complaints; fresh root paste applied for wound healing.
*Prunus persica* Stokes*.*	Fresh leaf paste applied on head for 2–3 h.
*Psidium guajava L.*	Fresh leaves are chewed.
*Punica granatum* L.	Powder (50 g) of dried fruit peel taken orally with warm water for old cough; fruit juice (50 ml) given twice a day to anemic patient.
*Ranunculus repens* L.	Root paste (50 g) applied for boils, and 30–50 ml filtered root extract (juice) is given twice a day against intestinal pain.
*Rephanus sativus* L.	Vegetable prepared from fresh leaves and root as salad.
*Rheum emodi* Wall.	Decoction of 100 g root with warm water taken orally (10 ml) for fever twice a day; root paste applied on wounds.
*Rhododendron arboreum* Smth	Juice extracted from fresh flowers
*Rosa moschata* Hermm.	Fresh leaf paste is applied on cuts, wounds, and boils; water extracted from fresh flowers used in eye diseases.
*Rubia cordifolia L.*	Root decoction with water given orally (1–2 TS) against fever twice a day to children (5 months–10 years)
*Rubus ellipticus* Smith.	Decoction (10 ml) of 100 g roots with water taken orally against fever and stomach ache for 5 days.
*Saussurea costus* (Falc.) Lipsch	Decoction of root (50 g) with water given against dysentery for 3–5 days twice a day; root powder (50 g) taken orally with boiled water in fever, cough, and stomach ache.
*Setaria italica* L.	*Koni ka Jaula* (an indigenous dish) prepared from seeds cooked with water.
*Silene vulgaris* (Moench) Garcke	Root decoction (10 ml) with warm water given against fever for 3 days; 1 TS is used for removal of *Ascaris* (*Juga*); leaves are used as a vegetable.
*Swertia* spp.	Juice of fresh leaves (100 g) given with boiled water 3 TS for 3–5 days for fever; *Panchang* (whole plant) is used after soaking overnight and taken (50–100 ml) orally in empty stomach for 15 days.
*Taraxacum officinale* Weber.	For snake bite: juice of whole plant with water taken orally (1–2 TS) thrice a day and applied on injured part for 1 week; mixture of 100 g roots with 9 seeds of black pepper, 1–2 l processed curd (*Mattha*), and 250 g paste of black soybean given to increase lactation in milching animals.
*Tegetus erecta* L.	Fresh leaf juice with water taken against fever (3–5 TS twice a day); leaf extract (2–3 drops) in ear infection; fresh leaf paste is applied for healing cuts and wounds.
*Terminalia chebula* (Gaertner) Retz.	Dried fruit powder (100 g) given orally with boiled water twice a day for 3–5 days in stomach ache; dried fruit crushed with water and given (1–2 ml) orally to children (3 months to 5 years) and small amount applied around the navel.
*Thalictrum foliosum* DC*.*	Fresh roots (50 g) soaked in rose water (100 ml) for overnight, filtered, and used as eye drop.
*Thymus serpyllum* L.	Paste of whole plant mixed with mustard oil gently applied on joints; whole plants juice (10 ml) mixed with honey (20 g) is taken orally for cough and asthma; broiled seeds (10–15 g) with warm water taken for digestive and stomach problems; leaves and seeds are used as spices and condiment.
*Trifolium repens* L.	Leaf paste (5 g) with water.
*Trigonella foemun-graecum* L.	Leaf juice is taken orally for curing obesity, indigestion, joints pain, and constipation; 25 g seeds are soaked overnight filter; the filtrate taken orally in empty stomach for gastric problems and diabetes.
*Urtica dioica* L.	Branches with leaves are gently rubbed on joints and muscles; fresh leaf twigs taken as vegetable; fine powder of dry leaf (5–10 g) dissolve in 50 ml water is taken orally in joints and muscular pain.
*Verbascum thapsus* L.	Fresh leaf paste applied on affected part for boils; 8–10 whole plants mixed with grass given mulching animals.
*Viola betonicifolia* J.E. Smith (Violaceae)	Paste of whole plant (fresh or semidry) applied on affected part for 1–2 weeks.
*Viola canescens* Wall. Ex Roxb	Fresh plants (30–50) given with grass for 1 to 2 weeks.
*Vigna mungo* L.	Paste prepared by grinding of 150 g seeds with water applied on the fractured part.
*Zanthoxylum armatum* DC	Seeds (100 g) boiled with water taken orally twice a day; seed bark used as a spices.
*Zingiber officinale* Rosc*.*	A piece (5–10 g) of broiled rhizome mixed with small amount of honey and chewed.

*FYM* farm yard manure, *TS* tablespoon

The community and *Vaidyas* identify each medicinal plant with a specific vernacular name. For example, *Bergenia ciliata* is identified by the community with a local name “*Pattharchatta*” (stone destroyer), and it is used in curing kidney stones. *Plantago ovate* is called “*Jonkpuri*” (jonk resembles worms) and is used in the treatment of *Ascaris* and other worms. *Viola betonicifolia* named “*Garur-Jadi*” (Garur means eagle), and it is used as an antidote to treat snake bites. Commonly, the community identifies a native name for species based on its local uses, ecology, physiology, anatomy, pharmacological activity, etc. [28].

**Fig. 6:**
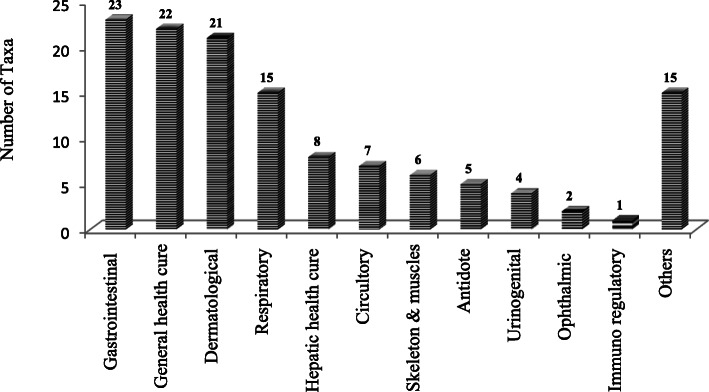
Distribution of medicinal plants in different ailments category

It was recorded that the species were used to cure a total of 12 major ailments (Fig. 6). Most species were used for curing gastrointestinal and general health disorders. It was followed by species used for treating dermatological and respiratory problems.

Lamiaceae has been the most dominating family for ethnomedicinal uses in the trans-Himalayan zone of Nepal [29] and Garhwal Himalaya in India as well [30]. Although the people in remote areas are still dependent on the traditional herbal cure system, it is being practiced by a few elderly people only. The young generation is not interested to take up this profession given minimal profit [3, 10, 12]. The common plant parts used in the present study are similar to other investigations [31–35]. The roots being the storage part of the plant contain valuable bioactive compounds [36]. Apart from the root part, leaves also contain a high concentration of health-beneficial secondary metabolites, phytochemicals, and essential oils, which contribute significantly to phototherapy or treatment of various health disorders [37–40]. The study reports 60% more species than reported earlier for the area under investigation [41–45].

### Quantitative analysis of ethnomedicinal information

The use value of important ethnomedicinal species was also calculated to depict the number of uses reported by the informants related to the utility of a species for a specific ailment or different ailments (Tables 1 and 3). Two forms of use reports were analyzed; the URc defines the use of a particular species to cure specific ailments as reported by all the informants, while URd reports the sum of all the uses for a particular disease/ailment. *Ocimum basilicum*, *Cannabis sativa*, *Citrus aurantifolia*, *Curcuma longa*, and *Setaria italica* have been top positioned in terms of use-reports and different ailments cured.

**Table 3:** Use value of important ethnomedicinal species of target area

Taxa	UR^**a**^	FC^**b**^	CI^**c**^	NDAS	Ailments categories (decreasing order)
*Ocimum basilicum* L.	97	88	0.97	5	Respiratory, general health care, and others
*Cannabis sativa* L.	94	63	0.94	6	Gastrointestinal, others, and antidote
*Citrus aurantifolia* (Christm) Sw.	94	38	0.94	6	Others, gastrointestinal, general health care, and respiratory
*Curcuma longa* L.	91	78	0.91	5	General health care, dermatological, and respiratory
*Setaria italica* L.	91	91	0.91	2	Dermatological
*Angelica glauca* Edgew.	89	44	0.89	4	Others and gastrointestinal
*Zingiber officinale* Rosc*.*	89	89	0.89	2	Respiratory
*Ajuga parviflora* Benth.	87	56	0.87	5	General health care, gastrointestinal, and urinogenital disorder
*Rephanus sativus* L.	87	87	0.87	1	Hepatic health cure
*Emblica officinalis* Gaertn.	85	35	0.85	6	Gastrointestinal, others, circulatory, and respiratory
*Glycine max* (L.) Merri	84	84	0.84	1	Hepatic health cure
*Plantago ovate* Forsk.	83	74	0.83	3	Gastrointestinal
*Ageratina adenophora* (Spreng.) King & H. Rob.	80	80	0.80	2	Dermatological
*Leucas lanata* Benth	80	80	0.80	1	Respiratory
*Picrorhiza kurrooa* Royle*.*	80	53	0.80	2	General health care and gastrointestinal
Artemisia martima L.	77	55	0.77	3	Dermatological
*Zanthoxylum armatum* DC	77	61	0.77	5	Others, general health care, respiratory, and gastrointestinal
Acorus calamus L.	74	55	0.74	3	Others and skeleton and muscles
Ajuga bracteosa Wall. ex Bent.	72	55	0.72	3	General health care, gastrointestinal, and urinogenital disorder
*Origanum vulgare* L.	71	31	0.71	5	Dermatological, respiratory, general health care, and others
*Punica granatum* L.	71	59	0.71	4	Respiratory, others, and hepatic health cure

*NDAS* no. of different ailment subcategories

^a^Total no. of use-reports of the taxon

^b^Use citation of taxa (the no. of informants that referred the taxon)

^c^CI = UR/*N*_t_, where *N*_t_ is the total no. of reported taxa

The usefulness of a species can be represented through its RFC value, which ranged 0.03 to 0.91 for different species (Table 1). Species with maximum RFC value were *Setaria italica*, *Zingiber officinale*, *Ocimum basilicum*, and *Raphanus sativus* which depict their higher use*,* while those with the least value comprised *Duchesnea indica* and *Thalictrum foliosum*.

The cultural importance index (CIs) specifies the distribution and importance of species in traditional herbal system, and the value ranged from 0.03 to 0.97. A total of 21 species have been identified as the most commonly used (Table 3). *Ocimum basilicum*, *Cannabis sativa*, and *Citrus aurantifolia* registered the highest cultural importance in the traditional herbal cure system. Low CI values specify that these species are either least used, or their use is declining in traditional herbal cure system [46].

An analysis of the informant consensus factor (*Fic*) for 12 broad treatment categories ranged between 0.92 and 1.0 (Table 4). The data revealed high homogeneity as per local people for all treatments. The immuno-regulatory category was assigned the value 1 due to the presence of only one taxon in the particular category. Apart from this, hepatic health care and urogenital categories obtained the value of 0.98 indicating well-defined criteria among the local population and non-random selection of species for the ailment category. *Asparagus recemosus*, *Glycine max*, *Hordeum vulgare*, *Polygonatum cirrhifolium*, *Punica granatum*, *Raphanus sativus*, and *Urtica dioica* not only used in hepatic health care but also provide nutritive benefits and warm potency, particularly at higher altitude areas. These species are commonly used in the daily food habit of the local community. Also, a higher value of *Fic* verifies the distribution of the different species used for a specific ailment. The urogenital category, with only 4 taxa included, comes second in terms of CI as there is a widely accepted notion of using these species for such disorders. The higher value of informant consensus factor for all the ailment categories also implies that the documented species are the most commonly used in traditional healing system.

**Table 4:** Informant consensus factor (*F*_ic_) and medicinal importance (MI) of ethnomedicinal plants

Ailments category	No. of taxa (***N***_**t**_)^**a**^	Frequency (%)^**b**^	No. of use reports (***N***_**ur**_)	Informant consensus factor (*F*_ic_)^**c**^	Medicinal importance (MI)^**d**^
Gastrointestinal	23	32.86	695	0.97	30.22
General health cure	22	31.43	524	0.96	23.82
Dermatological	21	30.00	617	0.97	29.38
Respiratory	15	21.43	402	0.97	26.80
Hepatic health cure	8	11.43	364	0.98	45.50
Circulatory	7	10.00	126	0.95	18.00
Skeleton and muscles	6	8.57	178	0.97	29.67
Antidote	5	7.14	83	0.95	16.60
Urinogenital	4	5.71	137	0.98	34.25
Ophthalmic	2	2.86	14	0.92	7.00
Immuno-regulatory	1	1.43	15	1.00	15.00
Other	15	21.43	377	0.96	25.13

^a^No. of species listed in several of the categories of medicinal usage

^b^Percentage of records on the total of 70 records

^**c**^*F*_ic_ = (*N*_ur_ − *N*_t_)/(*N*_ur_ − 1)

^d^MI = *N*_ur_/*N*_t_

The gastrointestinal ailments comprised of 695 use reports from the total categories with a medicinal importance index value of 30.22 (Table 4). Some most sought species in this category are *Cannabis sativa*, *Citrus aurantifolia*, *Angelica galuca*, *Ajuga parviflora*, and *Emblica officinalis.* These species are placed following their use reports mentioned during data collection. In the category of general health care, 22 species are being used with 524 numbers of use-reports and medical importance of 23.82. The species indicated with the highest number of use-reports are *Ocimum basilicum*, *Citrus aurantifolia*, *Curcuma longa*, *Ajuga parviflora*, and *Picrorhiza kurrooa* based on user reports. The dermatological category ranks third with 21 taxa in use and a use-report value of 617 and medicinal importance of 29.82. The main species employed for this category based on the use reports are *Setaria italica*, *Eupatorium adenophorum*, and *Artemisia martima*. Although the hepatic health cure category comprised of only 8 taxa, it has a medicinal importance index value of 45.50, which is highest of all the categories since the species used under the category are of daily usage and are often included in daily food products with nutritive values. The species include *Glycine max*, *Hordeum vulgare*, *Punica granatum*, *Urtica dioica*, *Polygonatum cirrhifolium*, etc. In other works carried out in Uttarakhand, they have reported these medicinal plants and use different plant parts in a different ratio to cure disease or aliments [16, 30, 31, 41–43, 45, 47–49].

A correlation analysis was done among RFC, CI, UR, number of species used in treating different ailments, informant consensus factor (*Fic*), and medical importance. No evidence of any correlation was observed in most of the parameters; a highly positive correlation was only observed in the number of taxa used and the number of use reports (0.963). Also, there has been a moderately positive correlation observed between *Fic* and RFC which is of no significance in the study as both the parameters have been described differently.

Some species are also used in ethnoveterinary purposes for curing domestic animals. *Ajuga parviflora* is used to cure throat infection, *Coriandrum sativum* against poison, and *Taraxacum officinale*, *Verbascum thapsus*, and *Viola canescens* to increase lactation in milking animals.

### The weakening of traditional ethnobotanical knowledge

**Fig. 7:**
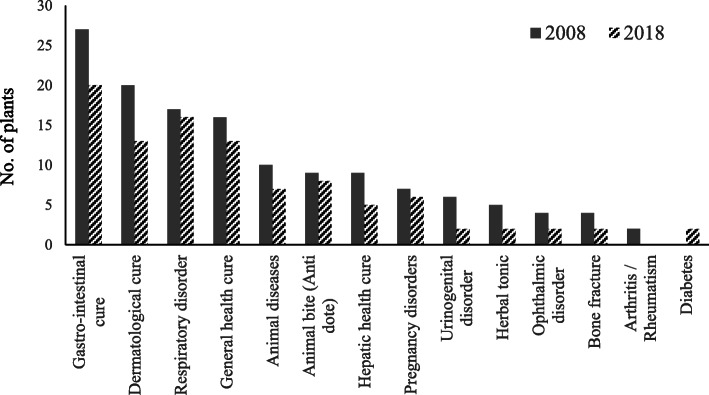
Past (2008) and present (2018) use of plants in traditional health care system

**Fig. 8:**
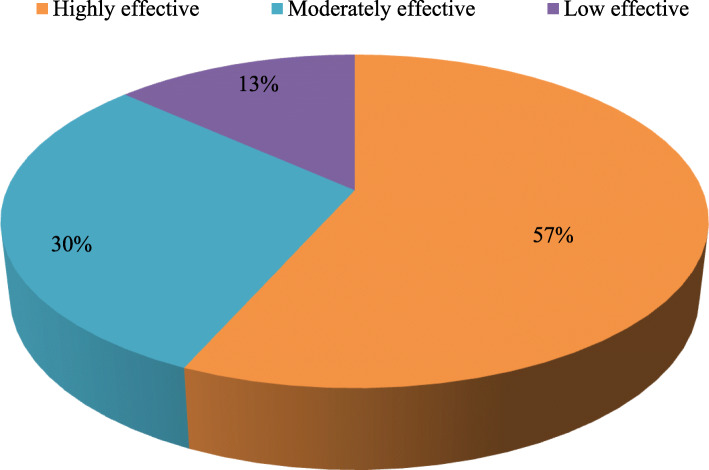
Community view points on effectiveness of traditional health care system

It is alarming to note that there has been a continued decline in traditional ethnobotanical knowledge in the target area (Fig. 7). An analysis of community perception on change in use pattern of medicinal plants in 2018 and a decade earlier (i.e., 2008) revealed that there is less number of species used for curing different ailments in recent years (Table 5). People are moving away from traditional herbal cure system, and the young generation has no interest in the traditional customs and values. Earlier, the people of remote areas preferred to consult with *Vaidyas* for primary healthcare, but in the last decade, since there is an increase in accessibility, availability, and affordability towards the allopathic medicinal system, the local community is also opting for such options. Despite that 57% of the total respondents believe that these plants are highly effective, 30% found moderately effective, while only 13% feel it less effective (Fig. 8). Interestingly, to cure selective diseases in children, such as *Juga* (removal of *Ascaris*), *Chupad* (heavy cough), and *Kasar* (constipation), still people prefer traditional cure systems as it has no side effects. During the study, it was observed that the *Vaidyas* do not share their knowledge; they believed that the treatment will not be effective if they share the knowledge with anybody. In the changing lifestyle and socioeconomic scenarios, most of the inhabitants are reluctant to live with their traditional heritage leading to the vanishing of the knowledge [58].

**Table 5:** Similarity between present and past ethnomedicinal uses of important species

Botanical name	Use reports in study area	Earlier use reports from Uttarakhand
*Aconitum heterophyllum* Wall.	Fever and vomiting	Fever, vomiting, and cough [21, 28, 51, 58]
*Acorus calamus* L.	*Inflammation and insect repellent	Arthritis, cancer, convulsions, diarrhea
	Sprain	Dyspepsia, epilepsy [41, 43]; sprain [50]
*Ageratina adenophora* (Spreng.)	Cuts and wounds	Cuts and wounds [31, 41]
*Ajuga bracteosa* Wall. ex Benth.	*Constipation	Fevers, diuretic [41]
	Diuretic, fever	
*Ajuga parviflora* Benth.	*Constipation, stone, throat infection in animal (*Galghotu*)	Headache, fever, stomach ache [51]
	Fever, stomach ache	
*Allium sativum* L.	*Joint pain (arthritis)	Muscular pain [43, 52]; ear pain [58]
*Angelica glauca* Edgew.	*Spices and condiment and herbal tea	Constipation, bronchitis, and stomach
	Stomach ache, vomiting	Disorders, vomiting [31, 43, 50]
*Artemisia martima* L.	Cuts, skin ailments, wounds	Skin ailments [51]
*Asparagus racemosus* Willd.	*Stimulant, tonic, and stomach ache	Leucorrhoea, headache, hysteria, ulcer, liver disorders [41, 43]
*Berberis asiatica* Roxb. ex DC	*Fever	Diabetes, jaundice [41]
	Diabetes	
*Bergenia ciliata* (Haw) Sternb	Urinary infection and stone	Fever, digestive disorders, skin diseases, urinary infection, and stone [16, 31]
*Cannabis sativa* L.	*Insect bite, stomach ache, purgative and laxative, warm effect in winters	Analgesic, cough, cold, sedative, narcotic, skin diseases [43]
	Carminative, constipation	
*Centella asiatica* L.	*Headache	Inflammatory infections, wounds [41, 43]
*Citrus aurantifolia* (Christm) Sw.	Cold, constipation, headache, herbal tea, source of vitamin “C,” and weight loss	Diarrhea, dysentery, fever, headache [53]
*Citrus hystrix* DC.	*Against poison, cold, removal of *Ascaris* (anti-parasitic)	Vomiting [52]
*Coriandrum sativum* L.	*Against poison	Stomachic and diuretic [43]
*Curcuma longa* L.	*Internal injury	Skin disorders, wound healing [43, 52]
	Cough, cuts and wounds, and cosmetics	
*Cynoglossum zeylanicum* Thunb. ex Lehm.	*Boils	Asthma, bronchitis, cough, vomiting [16, 54]
*Dactylorhiza hatagirea* (D.Don)Soo	Bleeding and wounds	Burns, cuts, checks bleeding [31, 41]
*Dioscorea deltoidea* Wall	Cough and cold	Cough, fever, urinogenital disorders [31, 41, 43, 51]
*Drymaria cordata* (L.) Willd. ex Schult	*Cough	Laxative [49]; bile complaints [51]
*Duchesnea indica* (Andrews) Focke	*Burns and removal of burn scars	Diarrhea, fever, leucorrhoea [54]; skin diseases [53]
*Emblica officinalis* Gaertn.	Diabetes, purgative and laxative, carminative, stomach ache, and source of vitamin “C”	Asthma, digestive disorders, hair fall [31]; dysentery, cholera, and jaundice [41, 51]
*Euphorbia prolifera* Ehrenb. ex Boiss	*Insect repellent	--
*Ficus palmata* Forsk.	*Cuts and wounds	Lungs diseases, skin diseases [43, 49, 51]
*Ficus roxburghii* Wall.	*Acidity, source of vitamin “C”	Laxative [49]
*Glycine max* (L.) Merri	*Jaundice	--
*Hedychium spicatum* Buch. Ham. ex Smith.	Anti-lice, cough, cosmetics, intestinal problems, purgative and laxative, carminative	Carminative, stomachic, liver complaints, fevers, vomiting, diarrhea, inflammation, snake bite [16, 41, 51]
*Hordium vulgare* L.	*Burns, warm, and nutritive effect	--
*Leucas lanata* Benth	*Cough	Cuts, to check bleeding, wounds [51]
*Mentha arvensis* L.	Stomach ache and vomiting	Diarrhea, stomach ache [51, 55]
*Micromeria biflora* Benth.	*Fever	Joints pain, worm infested wounds [41]
*Microtyloma uniflorum* (Lam) Verdc.	Stone	Stone [52]
*Momordica charantia* L.	Diabetes	Jaundice, diabetes [43]
*Ocimum basilicum* L.	Cough and cold, fever, herbal tea, warm effect in winters	Cough, cold, fever [16]
*Origanum vulgare* L.	Cough and cold, fever, herbal tea, and wounds	Cold, diarrhea, fever, indigestion, influenza, menstrual disorder [43, 51]
*Picrorhiza kurrooa* Royle.	Abdominal pain, fever	Anemia, asthma, blood troubles, inflammation, jaundice [41]; fever, stomach ache [31]; abdominal pain, cataract [50, 51]
*Plantago ovate* Forsk.	Constipation, digestive problems, and diarrhea	Constipation, dysentery, and diarrhea [41]
*Plantego lanceolata* L.	*Removal of stomach worm of domestic animals	Dyspepsia, sore wounds, dysentery, purgative, mouth disease, and chicks [41]
*Podophyllum hexandrum* Royle	Wounds	Purgative, cancer [41]; wounds [31]
*Polygonatum cirrhifolium* (Wall.)	*Blood purifier, cuts, tonic, and wounds	Anemia, fever, bronchitis, general debility [ 54]
*Polygonatum verticillatum* L.	Carminative and wounds	Aphrodisiac, gastric complaints, nervine tonic, wound healing [43, 51]
*Prunus persica* Stokes.	*Headache	Ear infection of children [31]; antipyretic, brain tonic [21]
*Psidium guajava* L.	Mouth blisters (astringent)	Mouth blisters [51, 59]
*Punica granatum* L.	*Anemia, cough, cold, source of vitamin “C”	Diarrhea, dysentery, piles [41]
*Ranunculus repens* L.	*Boils and intestinal pains (Nas Palatana)	--
*Rephanus sativus* L.	Jaundice	Jaundice [52]
*Rheum emodi* Wall.	Fever and wounds	Cuts, fracture, wounds [56]
*Rhododendron arboreum* Smth	Liver complaints, tonic	Heart tonic [31], stomach diseases [41]
*Rosa moschata* Hermm.	*Boils, cuts, eye diseases, wounds	Leucorrhoea, bleeding, pregnancy termination [16]
*Rubia cordifolia* L.	*Fever	Blood purifier, joints pain, leucorrhoea, cuts, wounds, insect sting [51]
*Rubus ellipticus* Smith.	*Fever and stomach ache	Blood pressure, diarrhea [41]
*Saussurea costus* (Falc.) Lipsch.	Cough, dysentery, fever, stomach ache	Asthma, cough, dysentery, fever [51, 55]; abdominal pain [58]
*Setaria italic* L.	*Chicken pox and measles	--
*Silene vulgaris* (Moench) Garcke	*Fever and removal of *Ascaris* (anti-parasitic)	Asthma, bronchitis [16]
*Swertia angustifolia* Buch.-Ham. ex D.Don.	*Skin ailments	Pneumonia, cold, cough, fever [51]
	Fever	
*Taraxacum officinale* Weber.	*Snake bite and to increase lactation in mulching animals	Headache, acts as a heart tonic and blood purifier [28, 58]
*Tegetus erecta* L.	*Ear infection, fever, and wounds	Muscular pain, piles, ulcer, wound healing [43]
*Terminalia chebula* (Gaertner) Retz.	Carminative, constipation, digestive problems, diarrhea, purgative	Asthma, digestive problems, diarrhea, purgative [16, 31]
*Thalictrum foliosum* DC.	*Eye infection (white-dot-cataract), insect repellent	Gastric trouble, used to control external parasites [41]
**Botanical name**	**Uses report in study area**	**Earlier uses report from Uttarakhand**
*Thymus serpyllum* L.	*Asthma, joint pain, spices, and condiments	Laxative, stomachic [41]; cough, epilepsy, itching, and skin diseases
	Digestive and stomach problems	Menstrual disorders, swelling [51]
*Trifolium repens* L.	*Headache and skin disease of dogs	Astringent [16]
*Trigonella foemun-graecum* L.	Carminative, constipation, diabetes, indigestion, joint pain, and obesity	Diabetes, rheumatism [16, 52]
*Urtica dioica* L.	*Joint pain, warm and nutritive effect	Skin diseases, boils [31, 41]; bone fracture [51]
*Verbascum thapsus* L.	*To increase lactation in milching animals	Cough, fever, rheumatism [41]; boils eye cataract [51]
	Boils	
*Viola betonicifolia* J.E. Smith	*Snake bite	Blood diseases, cough, fever, skin [57]
*Viola canescens* Wall. Ex Roxb	*To increase lactation in milching animals	Cough, cold, malaria, jaundice [43, 49]
*Vigna mungo* L.	*Fracture	--
*Zanthoxylum armatum* DC	Carminative, cough and cold, toothache, spices and condiments	Toothache [31]; constipation, gastric disorders [41, 43, 50]
*Zingiber officinale* Rosc. (Zingiberaceae)	Cough and cold	Asthma, cough, and cold [43]

*New ethnomedicinal use reports documented from study sites

## Conclusions

Community knowledge on the use and management of wild plant resources has always been integral to the survival, sustenance, and adaptation of human cultures [47, 53, 58]. This study revealed 70 medicinal plant species being used by the local marginal community of which 21 are the most extensively used species to treat various ailments. The significance of the traditional herbal healing system is highly relevant due to its effectiveness. It is cost-effective and based on local resources and still only means of cure for marginal communities in remote localities of Uttarakhand. With population growth and lack of health care, there is a need to adhere to the locally available resources to be utilized for general health care and provisioning of suitable side-effect free treatment to the communities. The community still uses these species; however, the level of use is decreasing because of upcoming modern allopathic based health care services. At the same time, there is also a decline in the number of local *Vaidyas* and herbal practitioners. This is because of increased access to modern hospitals and medicinal facilities in recent times. This possesses a significant challenge to the continuity of the traditional herbal cure system. The impoverishment of such knowledge may lead to an enormous loss to the scientific community. The ethnomedicinal knowledge and information provided in this study are of significant value for scientific validation, product development, conservation, and policy planners for sustainable management of medicinal plants and traditional herbal cure system. It is suggested to explore and establish linkage between traditional health practices and modern health care systems. It can be done by testing bioactive compound and biological activity of most preferred plant species and assessing the safety and efficacy of the local herbal formulation. Such an investigation may lead to many new and novel drug discovery. It is also recommended that the natural habitats of medicinal plants should be protected for the conservation of valuable gene pool and to control the exploitation of species. Since ethnomedicinal information is strongly linked to local livelihoods, culture, and environment, it is strongly recommended to further continue studying the subject to serve humanity with healthier and operative health care measures.

## Data Availability

The authors already included all data in the manuscript collected during the field surveys. The documented medicinal plant species were deposited at Centre of Socio-economic Development (CSED), GBPNIHE, Kosi-Katarmal, Almora, Uttarakhand.
